# Climatic influence on anthrax suitability in warming northern latitudes

**DOI:** 10.1038/s41598-018-27604-w

**Published:** 2018-06-18

**Authors:** Michael G. Walsh, Allard W. de Smalen, Siobhan M. Mor

**Affiliations:** 1 The University of Sydney, Faculty of Medicine and Health, Marie Bashir Institute for Infectious Diseases and Biosecurity, Westmead, New South Wales Australia; 2 The University of Sydney, Faculty of Medicine and Health, Westmead Institute for Medical Research, Westmead, New South Wales Australia; 3 The University of Sydney, Faculty of Medicine and Health, School of Public Health, Camperdown, New South Wales Australia; 4 The University of Sydney, Faculty of Science, School of Veterinary Science, Camperdown, New South Wales Australia

## Abstract

Climate change is impacting ecosystem structure and function, with potentially drastic downstream effects on human and animal health. Emerging zoonotic diseases are expected to be particularly vulnerable to climate and biodiversity disturbance. Anthrax is an archetypal zoonosis that manifests its most significant burden on vulnerable pastoralist communities. The current study sought to investigate the influence of temperature increases on geographic anthrax suitability in the temperate, boreal, and arctic North, where observed climate impact has been rapid. This study also explored the influence of climate relative to more traditional factors, such as livestock distribution, ungulate biodiversity, and soil-water balance, in demarcating risk. Machine learning was used to model anthrax suitability in northern latitudes. The model identified climate, livestock density and wild ungulate species richness as the most influential features in predicting suitability. These findings highlight the significance of warming temperatures for anthrax ecology in northern latitudes, and suggest potential mitigating effects of interventions targeting megafauna biodiversity conservation in grassland ecosystems, and animal health promotion among small to midsize livestock herds.

## Introduction

Anthrax is an important global disease of livestock that causes high animal mortality and is associated with spillover to humans. Epizootic and zoonotic infection can be particularly impactful to pastoralist communities, with significant public health, veterinary, and economic consequences associated with outbreaks^1^. Wild and domestic ungulates are typically infected when they ingest the anthrax spores during grazing or browsing. Edema, hemorrhage, and sudden death are common in animals, imposing a severe economic burden among pastoralists while also causing significant environmental contamination^2^. Human infection most frequently manifests cutaneous anthrax following direct contact with livestock or their fiber products. However, more severe disease can occur following consumption of raw or undercooked meat from infected animals, the inhalation of spores, or the development of septicemia following untreated cutaneous anthrax^2^. Several regions across the northern hemisphere are meso- or hyperenzootic for anthrax, including North America, eastern Europe, and central Asia. However, zoonotic transmission is typically well controlled in more affluent countries and less so in resource-poor countries, such that wide global disparity exists in human infection^1^.

Anthrax is caused by the spore-forming bacterium, *Bacillus anthracis*, which can remain viable across varied environmental conditions and over long periods of time^1,3,4^. The infection cycle of *B. anthracis* is complex, with the bacterium alternating between the vegetative replicating state in the host and the dormant sporulated state in the environment^1^. Sporulation occurs when the bacterium comes into contact with free oxygen in the air, that is, as exudates are released from the dead or dying animal. Spores remain dormant in the soil until they are ingested by another animal during grazing, at which point the spore germinates thus beginning the infection cycle anew. The bacterium prefers alkaline soils, but also occupies a spectrum of pH across the pedosphere^3,4^.The spores are resistant to heat and desiccation, enabling their persistence in the environment^1,4^. Moreover, the spores are subject to mechanical environmental displacement due to abiotic mechanisms such as flooding events^5–7^ and local slope^8^, or biotic mechanisms such as tabanid flies^9^. *B. anthracis* may also share a unique relationship with grasses and ruminants. *B. anthracis*-contaminated soil has been shown to enhance the establishment of grass species^10^, and these same species demonstrated significantly increased rates of growth in blood-contaminated soils^10,11^. The latter phenomenon is a common occurrence when ruminants succumb to the disease and exude profuse amounts of blood and body fluids into soil following death^1^. Thus, anthrax infection ecology includes important interaction between the pathogen, the host, the host’s herbivory and the broader environment.

Climate change has begun to manifest significant impact on terrestrial ecosystem structure, particularly in northern latitudes^12^. Soil composition, vegetation cover, the distribution of wild ruminant species, and the density and movement of domestic livestock are all shifting as temperatures rise globally and extreme weather events increase^13^. As ecosystems are challenged by climatic perturbation, new vulnerabilities may alter transmission cycles between hosts and pathogens^14–17^. Grasslands in the semi-arid steppe of the northern latitudes are fragile systems that may be particularly vulnerable to the influence of warming^12,14,18,19^. This may have important consequences for emerging diseases such as anthrax, as grasslands are important grazing zones for wild ruminants and the domestic livestock of pastoralist communities^20^. As the climate changes, so too may the distributions of these wild and domestic animals, thus potentially altering the interaction between pathogen, host and plant communities in northern biomes as described above. Furthermore, the modifying effects of climate may extend even farther north into the tundra biome, where recent outbreaks of anthrax in the Russian arctic have followed melting permafrost, devastating indigenous caribou-herding communities^21–23^.

The current study sought to investigate the influence of temperature anomalies on global anthrax suitability in northern latitudes. A machine learning algorithm was used to model anthrax outbreaks as a function of climate, soil composition, pasture land use, livestock density and wild ungulate species richness. It was hypothesized that areas of increasing average annual temperature would manifest higher suitability than areas without anomalous warming.

## Methods

Anthrax occurrences were acquired from the World Animal Health Information System (WAHIS) web portal[WAHID], which is the biosurveillance archive maintained by the World Organization for Animal Health (OIE)^24^. Each documented occurrence reported the location, date, type of livestock affected, and the number of infected animals. Because the objective of this study was to explore the influence of temperature anomalies on anthrax suitability in the temperate, boreal, and arctic regions of the global North, only outbreaks occurring at or north of 25°N latitude were included. Seventy-six anthrax submissions, reporting on 82 geographically unique outbreaks, were made to OIE between January, 2005 and December, 2016 in this northern extent. Due to a lack of reporting to OIE in the United States (US), Canada, and China, the OIE data were supplemented with additional data obtained from the ProMED-mail electronic surveillance system. This system is maintained by the International Society of Infectious Diseases and provides archival documentation of formal and informal reports of infectious diseases^25^. This latter source has been shown to provide good coverage of zoonotic disease events in North America and demonstrates a low rate of reporting error^26^. An additional 63 outbreaks were identified in ProMED-mail, two-thirds of which were from the US (n = 17) and Russia (n = 25). The geographic coordinates for all events reported in both OIE and ProMED-mail were obtained in Google Maps as the centroid of the reported municipality. A total of 145 anthrax outbreaks between 2005 and 2016 were available for analysis.

The temperature anomaly data product was obtained from the NASA Earth Observation (NEO) data repository for satellite imagery as a 0.5 degree raster for each year from 2005 to 2016^27,28^. Temperature anomaly for any given year between 2005 and 2016 represents the extent of annual temperature divergence (in degrees Celsius) from the homogeneity-adjusted, weighted-average annual temperature during the period 1951-1980^28^. The latter period was selected as the baseline, consistent with the detailed study of global temperature change initiated by the Goddard Institute for Space Science (GISS) in 1978. GISS used a three decade period as the baseline climate interval because 30 years are the accepted length of observation required to record one temporal unit of climate^28^. This specific period (1951–1980) was used because reliable global temperature estimates were not available prior to 1950^28^. In the current investigation, temperature divergence was captured as a global raster for each year under anthrax surveillance (i.e. 2005 to 2016). Mean temperature anomaly was then calculated across the 2005–2016 time period to represent the average warming trend between two serial climatic time points (point 1 = 1951–1980; point 2 = 2005–2016).

In addition to exploring the influence of warming anomalies between the present and the recent past, we also wanted to predict future anthrax suitability based on projected future warming. Mean annual temperature projected for the year 2050 was constructed by the Coupled Model Intercomparison Project Phase 5 (CMIP5) and acquired from the WorldClim data repository^29^. CMIP5 is a collaborative climate modeling project incorporating twenty modeling groups to predict future climate under the representative concentration pathways (RCPs) adopted in the fifth report of the Intergovernmental Panel on Climate Change^30,31^. The mean of the 19 climate models produced by CMIP5 was computed to obtain the future global climate estimate. Projected warming was evaluated at RCPs representing 4.5 W m^−2^ and 8.5 W m^−2^ radiative forcing due to CO^2^ concentrations because these intermediate and high emission scenarios, respectively, have become the standard for modeled climate change projection^32^. A raster of projected warming anomalies was used with the estimated Maxent model described below to predict anthrax suitability in 2050.

The Priestley-Taylor α coefficient (P-Tα) was used as a robust indicator of water-soil balance in the landscape^33,34^. This coefficient is the ratio of actual evapotranspiration to potential evapotranspiration. It represents water stress in each 1 km^2^ by capturing both water availability in the soil and water requirements of the local vegetation and contrasting this with solar energy input. Thus the measure is a robust estimate of environmental water stress through soil-water balance. A global raster for P-Tα was retrieved from the Consultative Group for International Agricultural Research (CGIAR) Consortium for Spatial Information. The ratio is dimensionless and ranges from 0 (high water stress) to 1 (low water stress)^35^.

The Global Soil Dataset for Earth System Modeling was sourced for soil pH and organic carbon content, which is based on an improved protocol of the Harmonized World Soil Database^36^. The resolution of these two rasters is 5 arc-minutes (approximately 10 km).

Rasters quantifying the proportion of pasture land^37,38^ and mammalian species richness^39^ were obtained from the Socioeconomic Data and Applications Center (SEDAC) repository at 5 arc-minutes and 30 arc-seconds, respectively. The latter was used to construct a profile of wild ungluate species richness comprising the Cervidae, Bovidae, Equidae, Camelidae, Antilocapridae, Suidae, and Tayassuidae families. Livestock densities for cattle, sheep, and goats were acquired from the Gridded Livestock of the World (GLW) as 30 arc-second rasters^40^.

The human footprint (HFP) was used to weight the sampling of background points to correct for potential reporting bias in anthrax presence points (see modeling description below).The HFP was quantified using data obtained from SEDAC^41^. The HFP was calculated in two stages, based on the methods by Sanderson *et al*. (2002)^42^. First, the human influence index (HII) was constructed. The HII measures the impact of human presence on the landscape as a function of eight domains: (1) population density, (2) proximity to railroads, (3) proximity to roads, (4) proximity to navigable rivers, (5) proximity to coastlines, (6) intensity of nighttime artificial light, 7) location in or outside delineated urban space, and (8) land cover. The domains are scored according to the level of human impact per geographic unit, whereby higher scores signify greater human influence. A composite index is then created by combining the eight individual domains. This composite ranges from 0, indicating an absence of human influence (i.e. a parcel of land unaltered by human activity), to 64, indicating maximal human influence in the landscape. The HII composite is subsequently normalized according to the 15 terrestrial biomes defined by the World Wildlife Fund to obtain the HFP. The normalization is represented as a ratio of the range of minimum and maximum HII in each biome to the range of minimum and maximum HII across all biomes, and is expressed as a percentage with a spatial resolution of approximately 1 km^2 ^^42^.

### Statistical Analysis

Maximum entropy (Maxent) machine learning was used to model anthrax suitability across the northern latitudes of the eastern and western hemispheres. Machine learning applications have gained wide use in modeling geographic suitability for many zoonotic infectious diseases^43–45^. Maxent in particular is analytically appealing because the model requires no specific functional form. Rather, homogeneity between the outcome and predictors is optimized based on data partition algorithms. Moreover, the locations of unknown (and unknowable) anthrax outbreak absences are not required by the Maxent algorithm to model the system^46,47^. The application of Maxent to ecological niche modeling is a popular implementation due to its robustness^48^.

The distribution of the climate, soil, land cover, livestock density, and wild ungulate species richness features described above were included in the Maxent model. The spatial scale of the analysis was 0.5° of arc, which is approximately 55 km, and so all covariates were aggregated to this scale. Therefore the resolution of predicted anthrax suitability is 55 km × 55 km. Since this study focused on the northern temperate, boreal, and arctic latitudes, environmental features were projected to the Lambert azimuthal equal-area to avoid the severe areal distortion in the high northern latitudes associated with other more common projections. Correlation was low among the covariates included in the model. All values of ρ were <0.6, and all but one (PT-α vs. soil pH: ρ = 0.57) were <0.4. We sampled 10,000 background points, which were weighted by the percentage of the human footprint in the landscape to adjust for potential reporting bias in anthrax surveillance^49^. The regularization parameter was selected at 1.0 to correct for overfitting. The Maxent model was trained using five-fold cross-validation, and performance was assessed as the area under the receiver operating characteristic curve (AUC). The environmental features were ranked according to their permutation importance in the model, which randomly permutes the values of the factors between background and presence points in the training dataset^46,50^. The best model, considering all available covariates, was selected using (1) a comparison of the test AUCs between the full and nested models, (2) a comparison of the Akaike information criterion as derived from fitting each model output to a Poisson point process to measure goodness-of-fit fit^51^, and (3) a jackknife variable selection procedure comparing each covariate’s lone contribution to the loss function to its effect on the loss function when the covariate is withheld from the model.

It has been observed that abiotic and biotic features may operate differently at different spatial scales in their relationships to disease processes^52^. As a sensitivity analysis the model was repeated at 1° resolution to evaluate whether anthrax suitability was robust to scale for both the abiotic and biotic features in the model.

Finally, the model was used to predict future anthrax suitability based on projected temperature increases using the averaged 2050 CMIP5 climate models as described above. Future suitability was based on climate projections at the 4.5 and 8.5 RCP scenarios. It is important to emphasize that this model was used to predict future anthrax suitability by updating temperature anomalies only using the future temperature projections. All other environmental features used to predict future anthrax suitability were based on present day values only and thus do not capture complete environmental feature projection as projected data do not exist for these latter features on a global scale.

The maxent function (dismo package; v. 0.9–3) was used to fit the model^47,53,54^. All analyses were performed using R statistical software version 3.1.3^55^.

### Data availability

All data generated or analyzed during this study are included in this published article (and its Supplementary Information files). Curated OIE and ProMED-mail data are available at the Figshare data archive (https://figshare.com/s/55a5c5e1b8e11085ea73).

## Results

**Figure 1:**
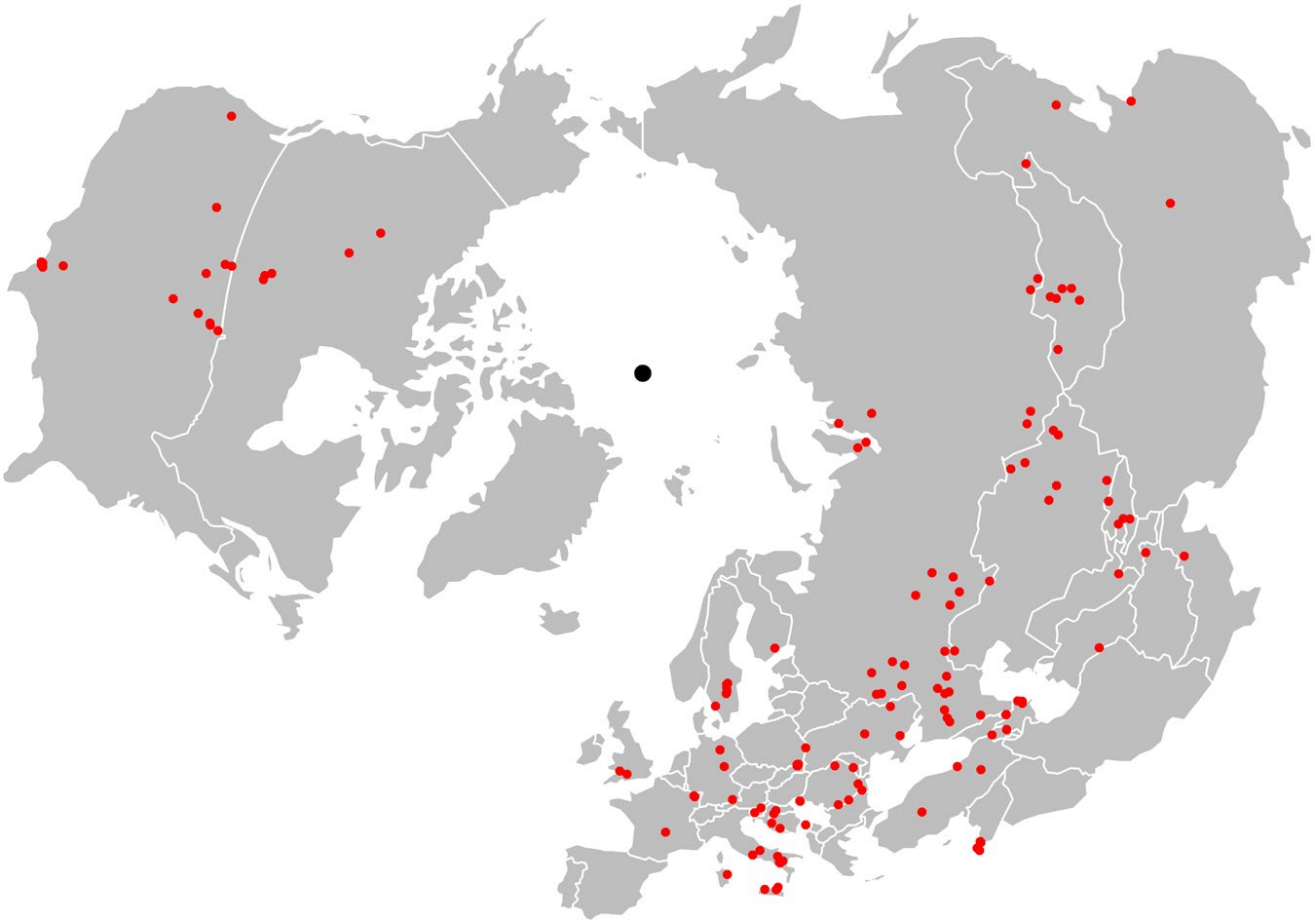
Distribution of anthrax outbreaks as documented by the World Organization for Animal Health (OIE) and Pro-MED mail surveillance mechanisms between 2005 and 2016. For orientation with the Lambert azimuthal equal-area projection of the map, the North Pole is represented as the central black dot. All maps created in R (v. 3.3.1)^55^.

The distribution of anthrax outbreaks in the temperate, boreal, and arctic regions of the northern hemisphere is presented in Fig. 1. The distribution of the climate, soil chemistry and pasture cover, and livestock and wild ungulate species richness features are presented in Supplementary Fig. S1.

**Figure 2:**
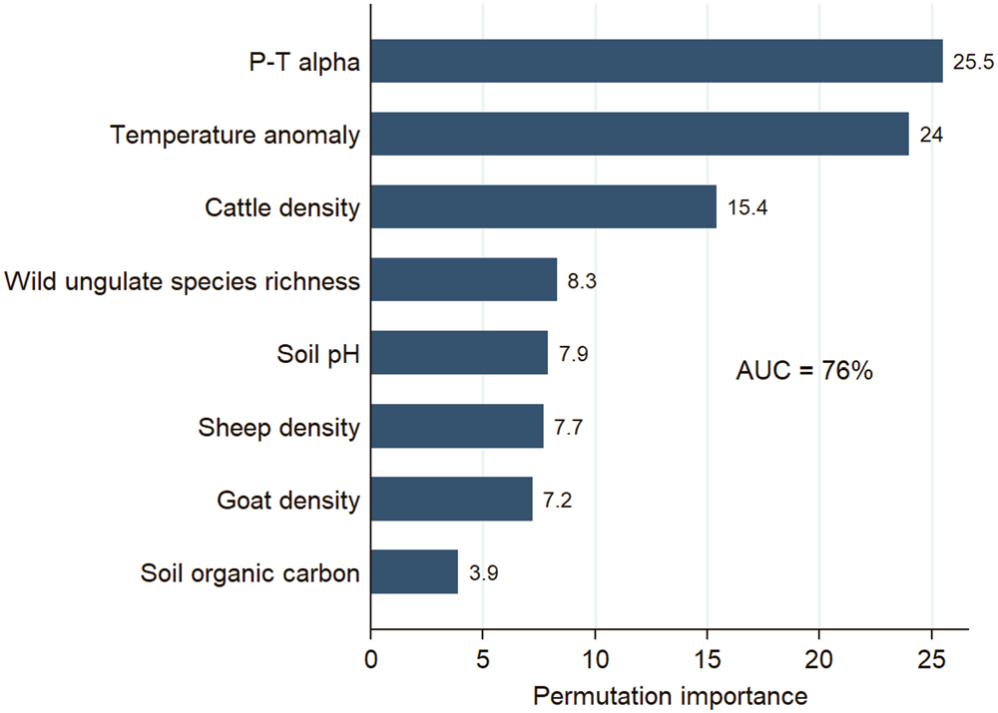
Environmental feature ranking according to each feature’s permutation importance in the Maxent model. The area under the curve (AUC) is reported as a percentage.

The best model comprised climate, soil chemistry, and animal features, but excluded pasture cover (Model 6 in Supplementary Table S1; Supplementary Fig. S2). Climate was associated with anthrax suitability, whereby warming temperature anomaly (permutation importance = 24%) and the P-T α for water-soil balance (permutation importance = 25.5%) together contributed 49.5% in permutation importance (Fig. 2). Livestock densities (30.3%, combined) and wild ungulate species richness (8.3%) were also important predictors of anthrax suitability. While not dominating features, soil pH and organic carbon content nevertheless were still impactful in the model, collectively contributing almost 12% to the loss function. Model performance was reasonable with the AUC equal to 76%.

**Figure 3:**
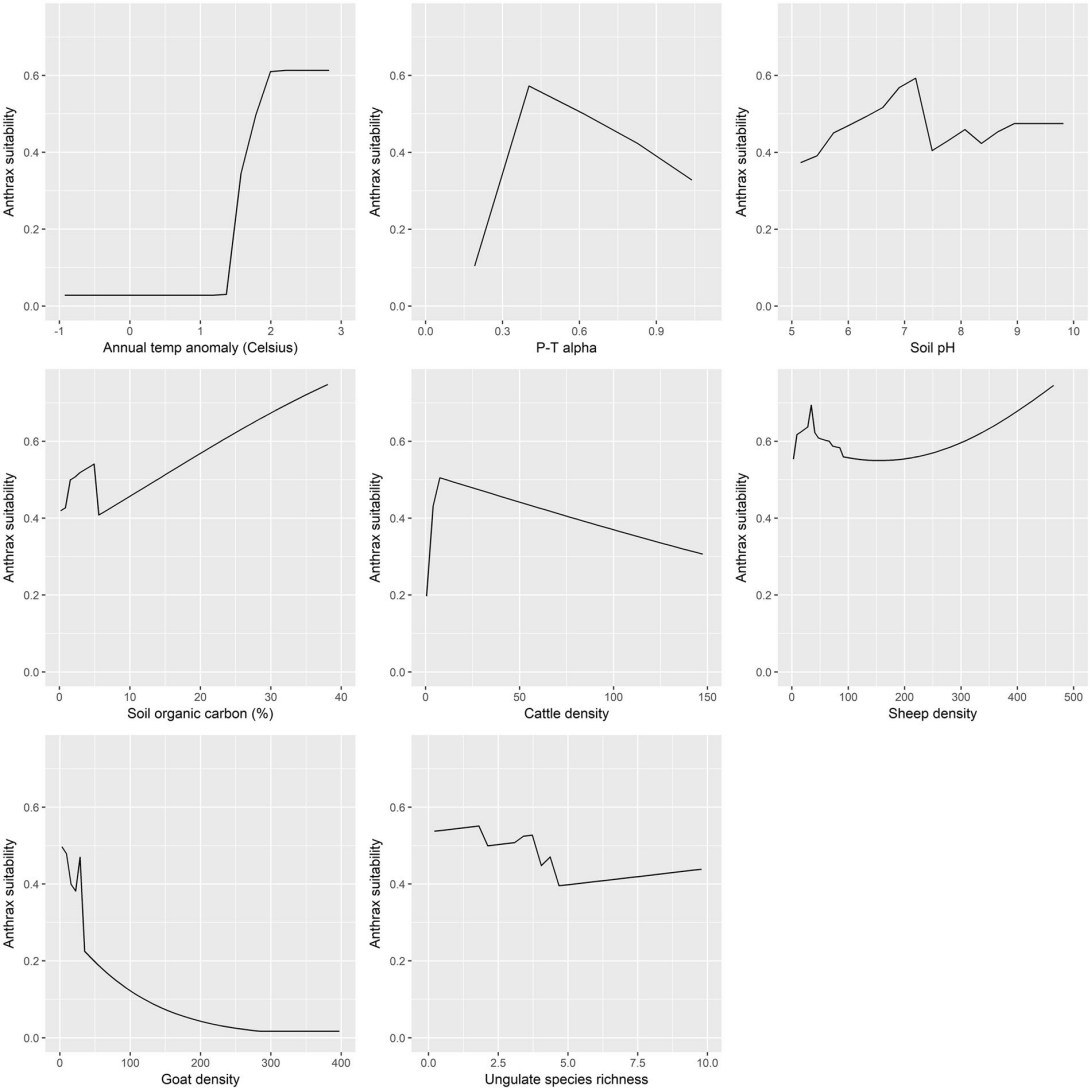
Response curves showing the functional relationships between each feature and anthrax suitability.

The response curves showing the functional association of each environmental feature conditional on all others are presented in Fig. 3. Predicted anthrax suitability increased dramatically in areas of large anomalous warming. Suitability was high in the areas of mild to moderate water stress with P-Tα peaking at 0.38, which is typical of semi-arid steppe landscapes. However, suitability was low in areas of extreme water stress or in areas absent of water stress. Soil pH in the range of 5–7.5, and soils with higher organic carbon content, were associated with greater suitability. Cattle, sheep, and goat density were influential in the model, however cattle and goat density were associated with greatest risk among smaller herds while sheep demonstrated elevated risk at low and high density. Finally, increasing wild ungulate species richness was associated with decreasing anthrax suitability.

**Figure 4:**
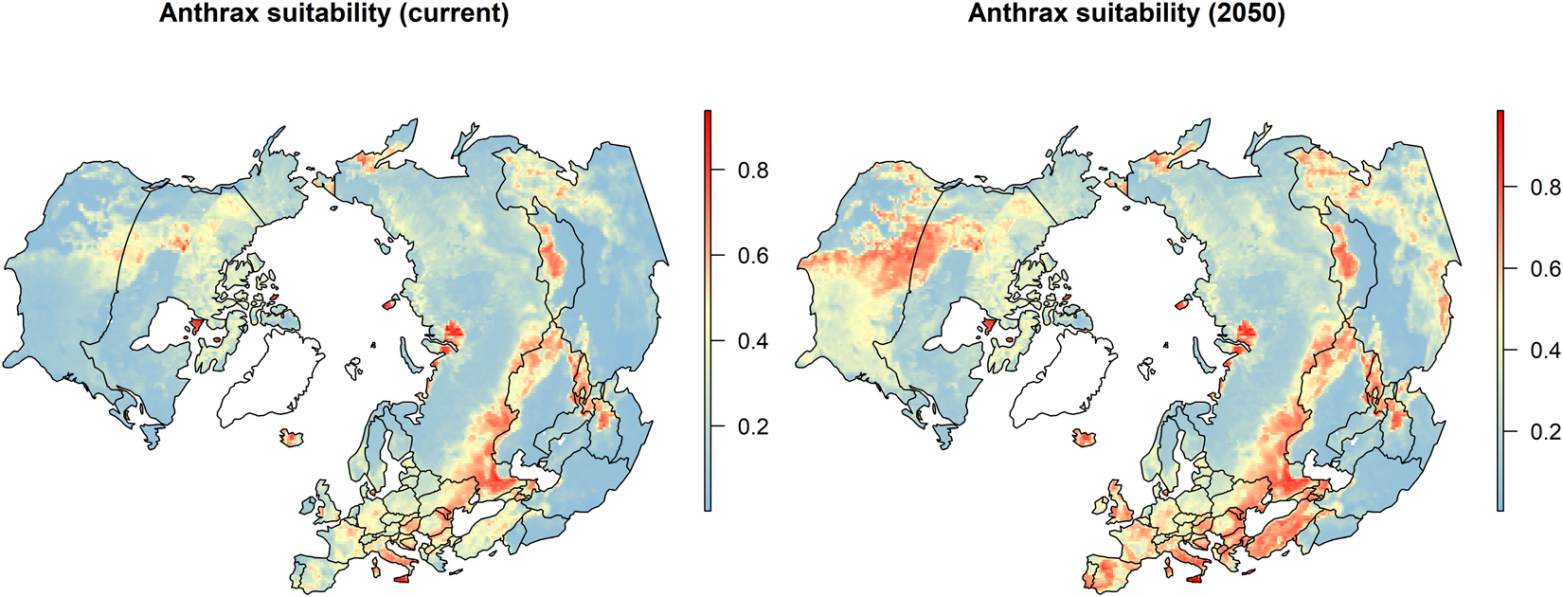
Predicted anthrax suitability based on current warming anomalies (left panel), and future anthrax suitability based on projected anomalies in 2050 using a representative concentration pathway of 4.5 W m^−2^ radiative forcing due to CO^2^ concentration (right panel). Greenland is shown in outline only as there was insufficient data for each feature to model the suitability. All maps created in R (v. 3.3.1)^55^.

The predicted anthrax suitability in the northern latitudes is presented in Fig. 4 (left panel). In the eastern hemisphere, an intercontinental anthrax suitability corridor emerged that extended from Eastern and Mediterranean Europe across the steppe of Russia and central Asia and into northwestern China. In addition, two narrow high risk corridors extended from the intercontinental anthrax belt southward down the eastern and western boundaries of central Asia. There were also prominent areas of high suitability in some areas of the arctic and sub-arctic North, particularly in northern Siberia, Iceland, and parts of Scandinavia and the UK, although these were not as extensive as the steppe regions to the south. A marked corridor of anthrax suitability also emerged in the western hemisphere, running from north to south, and corresponding to the landscape configuration of steppe country in North America. The areas of greatest risk were in south-central Canada and the north-central United States. However, there were some areas of high suitability identified in the high Arctic. The sensitivity analysis showed no meaningful differences in anthrax suitability when evaluated at 1^0^ resolution (Supplementary Fig. S3). Anthrax suitability predicted by 2050 identified the same general regions of the temperate, boreal, and arctic North, but risk had increased and expanded substantively in many areas, particularly in North America, southern Central Asia, Mediterranean Europe, and China (Fig. 4; right panel). This was similar to projections using the high RCP scenario (8.5 RCP; Supplementary Fig. S4)

## Discussion

This investigation is the first to describe anthrax suitability across the temperate, boreal, and arctic North of both the eastern and western hemispheres. We found that climate and the distribution of domestic livestock and wild ungulate species richness were important predictors of suitability. Warming annual temperature anomalies were associated with increasing suitability, as was a water-soil balance that favored mild to moderate water stress. This is the first study to show an increase in anthrax risk across the northern latitudes associated with a warming climate, an association that was highly influential to predicted suitability. Moreover, anthrax suitability is predicted to expand and increase within a relatively short period of time (~30 years) with continued warming. The effects of livestock densities and soil composition notwithstanding, these results highlight the potential effects of rising temperatures and moderate water stress on anthrax risk in northern latitudes.

While this is the first study to explore anthrax suitability across extensive northern latitudes, our results identified a risk profile that was generally in geographical agreement with other regional suitability studies^56–58^. Anthrax suitability identified by the current study corroborated the findings of two previous studies in the United States^56^ and Kazakhstan^57^, although the suitability predicted for Texas in the current study was not as strong as that predicted in the previous United States model. Both of these earlier studies also included measures of climate in their models, however only the latter incorporated measures of climate change. A third study of anthrax risk in China was less convergent with our current findings^59^. The two studies did agree on predicted risk with respect to the northeastern and northwestern regions of China, however they diverged on the predicted risk for central China. These differences are not surprising given that the previous study used surveillance data aggregated at the county and province level and also generalized several disparate measures of climate.

In the current study, areas where recent annual temperatures (2005 to 2016) exhibited warming divergence from past annual temperatures (1950 to 1980) were more suitable to anthrax outbreaks than those with no change or cooling divergence. These areas of anomalous warming were most pronounced in the higher latitudes. There are several mechanisms by which warming may influence suitability across intercontinental scales of varying ecosystem structure. First, warming trends in the Arctic have been associated with substantial permafrost melt^60–62^, which may operate directly in the infection cycle by thawing wild or domestic ruminant carcasses, or their previously frozen tissues or body fluids, and subsequently releasing spores into the soil^22,63^. When grazing livestock or wildlife subsequently ingest these spores, the spores can germinate and thus continue the infection cycle. This phenomenon has been documented at the animal burial sites of previous epizootics^21,23,64^. The warming Arctic, with its attendant changes in the presence of ice and water in the landscape, has also been shown to significantly modify the migration routes of indigenous pastoralist communities^65^, as well the migratory patterns of wild ungulate species such as caribou^66^. This may also lead to novel environmental exposures as previous barriers disappear and emerging bottlenecks or range expansions appear. Second, warming in semi-arid steppe or shrubland may increase dry conditions that may be favorable to anthrax spore dissemination in the middle and southerly northern latitudes, particularly when summer droughts are punctuated with intermittent precipitation^5–7^. The current study lends further support to the favorability of relatively dry conditions as anthrax suitability appeared to be optimized in areas with a mild to moderate degree of water stress, which is typical of the grasslands and steppe of eastern Europe, north-central Asia and north-central North America^67^. Dry soils may favor mechanical dissemination of spores by intermittent precipitation events^68^. Third, hot weather may modify host susceptibility by decreasing immunocompetence^69–71^. Therefore, warming temperatures and dry conditions may enhance the transmission cycle of *B. anthracis* by simultaneously opening new chains of transmission, promoting mechanical motility through the environment, or suppressing host immune function. While the climatic features in the current study support an infection ecology modulated by warming annual temperatures and moderate soil-water stress, none of the potential mechanisms described above were directly observed in the current study and, as such, definitive pathways describing anthrax epidemiology must await interrogation by field studies of livestock, wildlife, and the environment.

The distribution of animals was influential to anthrax suitability, with livestock density and wild ungulate species richness accounting for 38.6% combined permutation importance. Small herds were associated with greatest risk among cattle, while sheep predicted anthrax suitability in low and high density. This may reflect greater adoption of preventive measures (i.e. vaccination) in areas supporting large, or industrial, cattle industries^72,73^, whereas such prevention may be more lacking in general in sheep husbandry. Increasing ungulate species richness decreased suitability for anthrax, which is consistent with the view that high biodiversity has a diluting effect on pathogen transmission^74,75^. Nevertheless, the functional relationship between anthrax suitability and ungulate biodiversity was not particularly strong as depicted in the response curve (Fig. 3), suggesting that the current results are not comprehensive enough to describe how ungulate biodiversity operates with respect to disease risk in northern latitudes.

Soil organic carbon and pH have long been recognized as important properties in the infection ecology and transmission cycle of *B. anthracis*^3,4^. High organic carbon, and pH in the range of 6-7, delineate the Mollisol and Chernozem soils of the prairies of North America and the steppe of eastern Europe and central Asia, respectively, which are typically favorable to anthrax sporulation in northern latitudes^4,68^. These soils tend to be rich in calcium, which can further promote spore germination under the right environmental conditions^3,76,77^. Moreover, these soil profiles support rich grassland ecosystems and agricultural production^78^, which is likely reflected in the influence of livestock densities on anthrax suitability. Thus, while soil composition was not as influential as climate and livestock density, the current study does reinforce the pedological and edaphological paradigm for *B. anthracis* across both the western and eastern hemispheres.

This study has some important limitations. First, the documented anthrax outbreaks included in this study are derived from the OIE and ProMED surveillance systems, which do not necessarily capture all anthrax outbreak occurrences and which may be more responsive in areas with better veterinary services and reporting infrastructure, thus potentially leading to reporting bias in the identified anthrax locations. Moreover, such bias is likely to reflect global economic disparity since resource-poor countries or locales are typically those unable to afford the cost of such animal health infrastructure. We attempted to correct for such reporting bias by selecting background points weighted by the presence of the human footprint as a proxy for reporting infrastructure. Nevertheless, we must concede that these data may not be representative of the total anthrax outbreaks occurring in northern latitudes over the time period under study, but rather reflect geographies wherein a basic level of animal health service and disease reporting has been achieved. Second, the scale of the study is coarse. While this is unlikely to be of substantial influence to environmental features operating at small (i.e. coarse) spatial scale, such as temperature, soil composition, and wildlife species range, it would most certainly be influential to features operating at large (i.e. fine) scale, such as subtle changes in topographic slope and water drainage, or the distribution of tabanid flies as mechanical vectors. As such, we were not able to investigate the additional influences of hyper-local features such as water pooling, which has been previously identified as an important delimiter of anthrax risk due to spore accumulation through water dispersal^8^, or the ecological niche of tabanids despite convincing evidence that these may be important to anthrax transmission in some regions^9^. Third, temperature anomaly was assessed over a relatively recent time frame (i.e. change between 1950–1980 and 2005–2016), and therefore assumes as its baseline a temperature regime that was already likely undergoing warming trends. Nevertheless, we would expect this to dilute the effect of global warming on anthrax suitability. However, given the strong influence that was still apparent in the current analysis, it would seem that temperature increase is impactful to the anthrax suitability even in the short term. As mentioned previously, it is important to note that future anthrax suitability is based on projected temperature anomaly, while such projected data was not available for the other environmental features. As such, the model predictions for future anthrax suitability lack the nuance offered by a fully future-projected environmental space.

In conclusion, this investigation revealed the novel finding that, mean global temperature increases between 1950–1980 and 2005–2016 identified high anthrax suitability in the northern latitudes during the early decades of the twenty-first century. Continued warming in northern latitudes predicted expanding anthrax risk. In addition, livestock density and wild ungulate species richness were also associated with anthrax suitability. These results provide the first evidence that climate change may influence the risk of this impactful zoonotic disease across a large extent of the northern latitudes. Additional findings from this study suggest that interventions targeting animal health promotion among small to midsize livestock herds (e.g. vaccine promotion among pastoralist communities) may reduce the overall suitability to future outbreaks, possibly mitigating some of the effects of climate change. The association between anthrax suitability and ungulate species richness is also intriguing for the potential One Health implications of megafauna biodiversity conservation in grassland ecosystems, however the current results are not yet sufficient to make a claim for specific conservation intervention strategies and will require validation from more localized studies to confirm the possibility of real benefit.

## Electronic supplementary material

Supplementary Information
