# Supplementary material for: Climatic influence on anthrax suitability in warming northern latitudes
Source: Sci Rep. 2018 Jun 18;8:9269. doi: 10.1038/s41598-018-27604-w (PMC6006314; doi:10.1038/s41598-018-27604-w)

Supplementary Information

Climatic influence on anthrax suitability in warming northern latitudes

Michael G. Walsh[1,2], Allard W. de Smalen[3], Siobhan M. Mor[1,4]

[1]The University of Sydney, Faculty of Medicine and Health, Marie Bashir Institute for Infectious Diseases and Biosecurity, Westmead, New South Wales, Australia [2]The University of Sydney, Faculty of Medicine and Health, Westmead Institute for Medical Research, Westmead, New South Wales, Australia, [3]The University of Sydney, Faculty of Medicine and Health, School of Public Health, Camperdown, New South Wales, Australia [4]The University of Sydney, Faculty of Science, School of Veterinary Science, Camperdown, New South Wales, Australia.

Supplementary Figure S1. The distribution of the climate and soil characteristics, pasture cover, livestock density, and wild ungulate species richness used in the modeling of the anthrax niche.

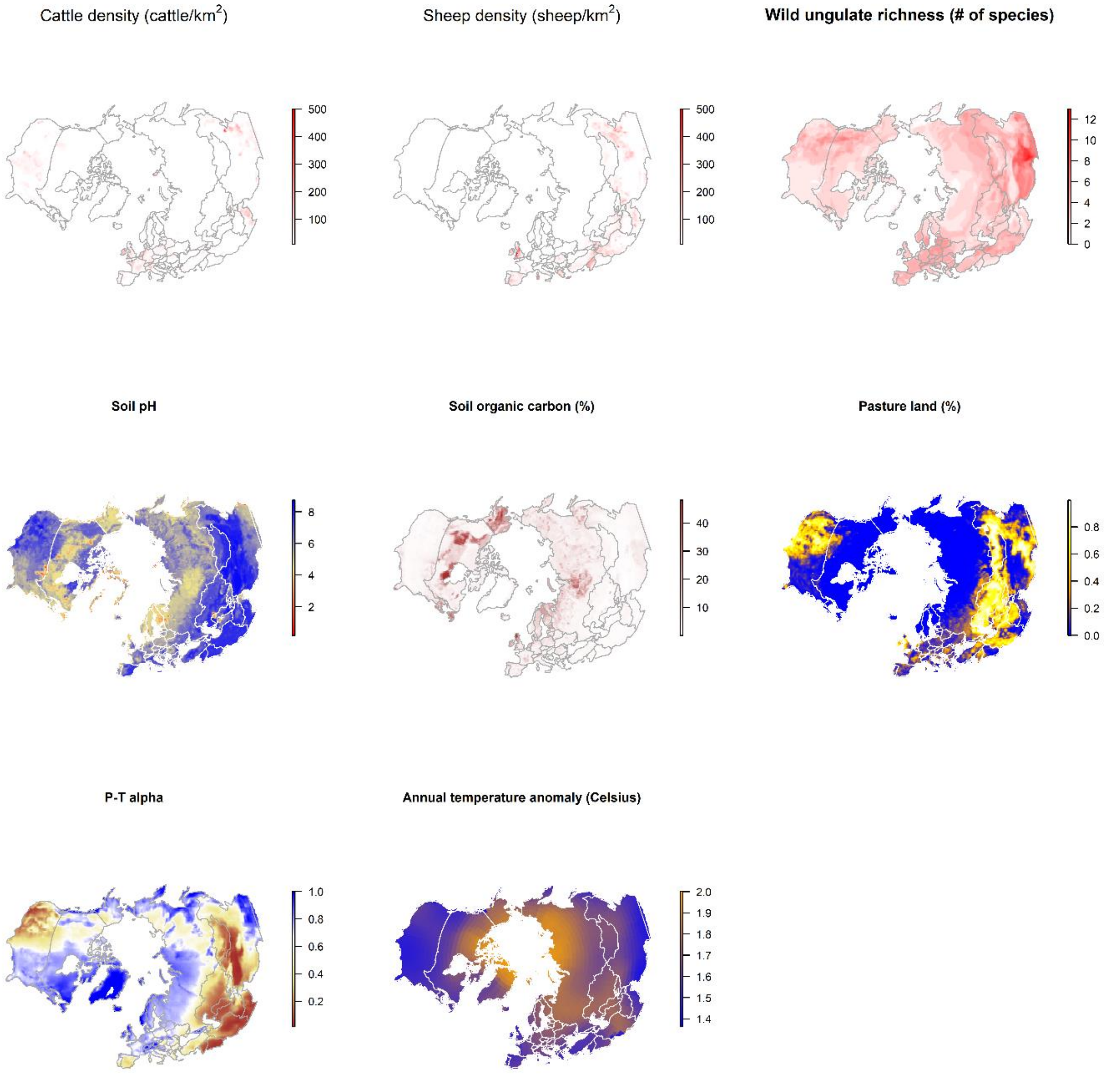

Supplementary Table S1. Model candidate evaluation comparing the full model with all covariates to reduced subset models. Comparisons are based on the Akaike information criterion (AIC) and the area under the receiver operating characteristic curve (AUC). The AUCs represent the mean from cross-validation.

Models	AIC	AUC (%)	AUC std. dev.
Model 1: Full model - All covariates included	1161.6	74.8	0.040
Model 2: Reduced - Livestock and ungulate species richness only included	1220.5	69.4	0.052
Model 3: Reduced - Model 1 covariates excluding livestock density and ungulate richness	1268.7	72.4	0.045
Model 4: Reduced - Soil chemistry only (organic carbon + pH) included	1298.0	68.4	0.058
Model 5: Reduced - Climate only (temperature anomaly + PT-α) included	1318.8	70.9	0.022
Model 6: Reduced - Model 1 covariates excluding pasture cover	1156.8	76.0	0.042

Supplementary Figure S2. Maxent model with the jackknife variable selection procedure comparing each covariate's lone contribution to the training gain (blue) to its effect on training gain when the covariate is withheld from the model (red).

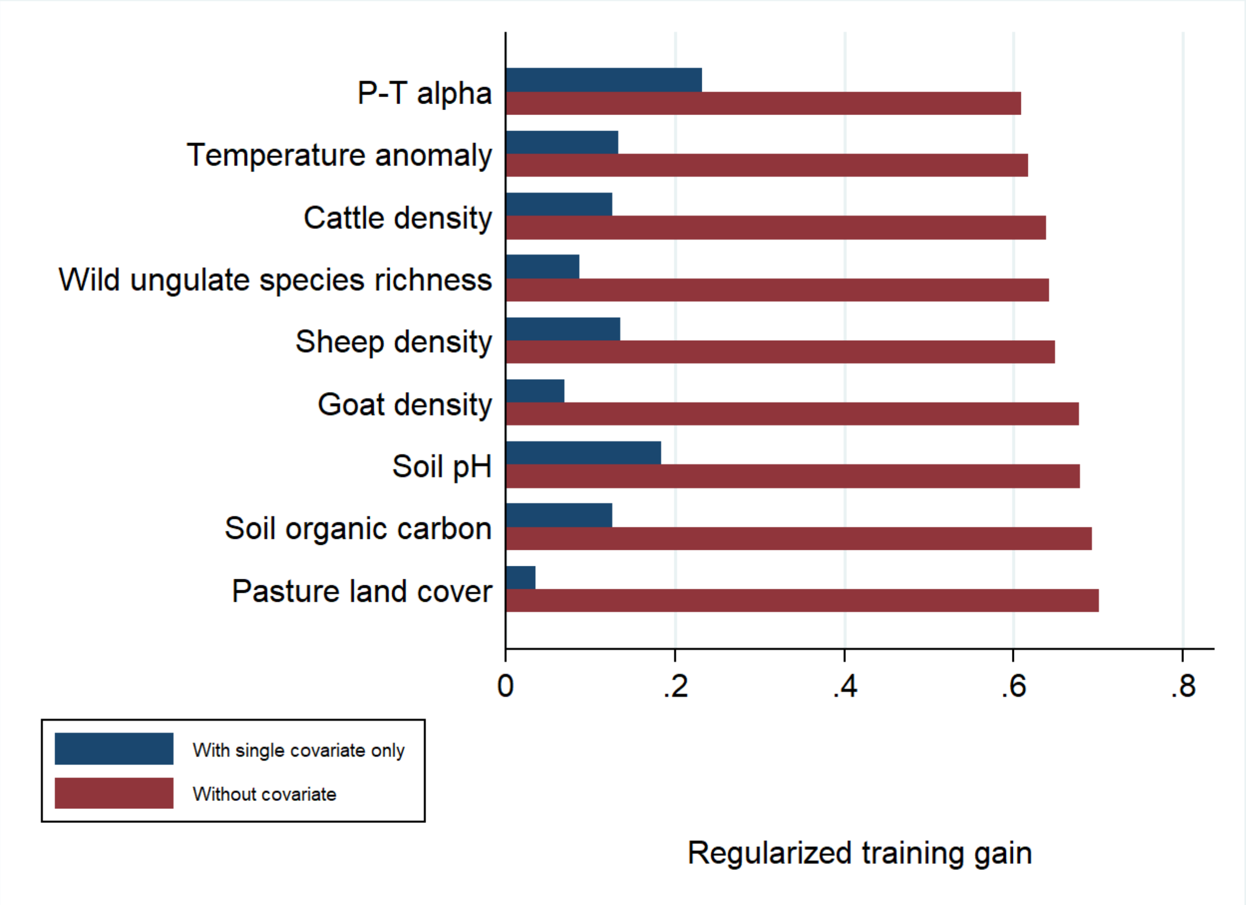

Supplementary Figure S3. Sensitivity analysis of anthrax landscape suitability evaluated at 1° resolution.

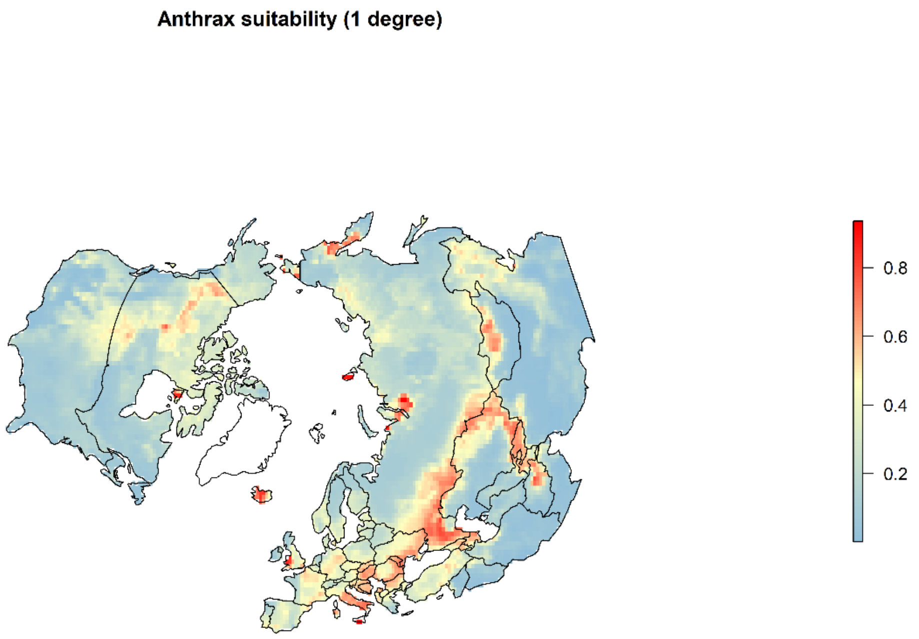

Supplementary Figure S4. Future anthrax landscape suitability based on climate projections at the 8.5 W m^{-2} representative concentration pathway scenario.

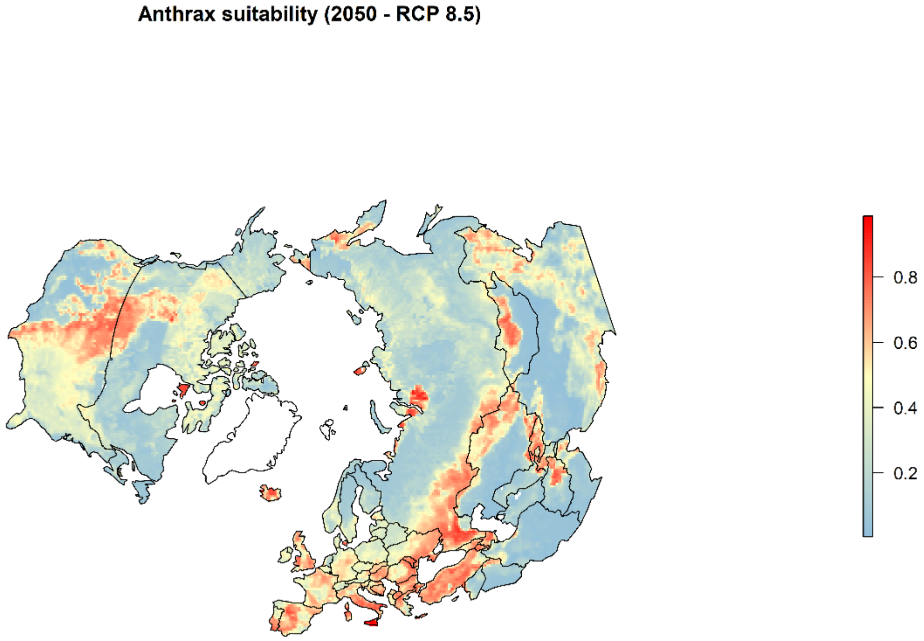

Supplement: Supplementary file 1 — Supplementary Information [file 41598_2018_27604_MOESM1_ESM.pdf]
